# Divergence of Phyllosphere Microbial Community Assemblies and Components of Volatile Organic Compounds between the Invasive *Sphagneticola trilobata*, the Native *Sphagneticola calendulacea* and Their Hybrids, and Its Implications for Invasiveness

**DOI:** 10.3390/genes15070955

**Published:** 2024-07-20

**Authors:** Hui Zhang, Shanshan Li, Sheng Zhou, Wei Guo, Ping Chen, Yongquan Li, Wei Wu

**Affiliations:** Scarce and Quality Economic Forest Engineering Technology Research Center, College of Horticulture and Landscape Architecture, Zhongkai University of Agriculture and Engineering, Guangzhou 510225, China; zhanghui@zhku.edu.cn (H.Z.); lss3433@163.com (S.L.); zhousheng@zhku.edu.cn (S.Z.); gwei717@163.com (W.G.); turfchen@163.com (P.C.); yongquanli@zhku.edu.cn (Y.L.)

**Keywords:** phyllosphere microorganisms, *Sphagneticola trilobata*, *Sphagneticola calendulacea*, hybrids, high-throughput sequencing, volatile organic compounds (VOCs)

## Abstract

Closely-related plant groups with distinct microbiomes, chemistries and ecological characteristics represent tractable models to explore mechanisms shaping species spread, competitive dynamics and community assembly at the interface of native and introduced ranges. We investigated phyllosphere microbial communities, volatile organic compound (VOC) compositions, and potential interactions among introduced *S. trilobata*, native *S. calendulacea* and their hybrid in South China. *S. trilobata* exhibited higher α diversity but significantly different community composition compared to the native and hybrid groups. However, *S. calendulacea* and the hybrid shared certain microbial taxa, suggesting potential gene flow or co-existence. The potent antimicrobial VOC profile of *S. trilobata*, including unique compounds like p-cymene (13.33%), likely contributes to its invasion success. The hybrid’s intermediate microbial and VOC profiles suggest possible consequences for species distribution, genetic exchange, and community assembly in heterogeneous environments. This hybrid deserves further study as both an opportunity for and threat to diversity maintenance. These differentiating yet connected plant groups provide insight into ecological and evolutionary dynamics shaping microbiome structure, species co-occurrence and competitive outcomes during biological exchange and habitat transformation. An interdisciplinary approach combining chemical and microbial ecology may reveal mechanisms underlying community stability and change, informing management of species spread in a globalized world.

## 1. Introduction

The phyllosphere, primarily consisting of the aerial parts of plant leaves, is a vast but often overlooked microbial habitat [1,2,3]. The global leaf surface area, estimated at 1,017,260,200 km^2^, is approximately twice that of the land surface. Assuming 10^6^ to 10^7^ bacteria per cm^2^ of leaf surface, the phyllosphere may host up to 10^26^ bacterial cells [1], with even higher numbers when including yeasts, filamentous fungi, and protists. The phyllosphere includes both epiphytic (leaf surface) and endophytic (leaf interior) microbiota. As epiphytic microbiota are more diverse and abundant [4,5], this study focuses on these surface-dwelling microbes. 

In recent decades, our understanding of plant–microbe interactions Has mainly focused on rhizosphere microbes. These diverse communities, often called the plant’s second genome [6,7], are crucial for plant health, while some beneficial microbes like plant growth-promoting rhizobacteria (PGPR), arbuscular mycorrhizal fungi (AMF) and nitrogen-fixing bacteria are well known, they represent only a small fraction of rhizosphere communities. Our understanding of rhizosphere microbe assembly and function remains limited [8]. The knowledge gap for phyllosphere microbe is even larger. However, studies on Arabidopsis, grapevine, and mustard plant *Boechera stricta* have revealed significant overlaps between key community members in the phyllosphere and rhizosphere [9,10,11]. This suggests that, phyllosphere microbes should be considered when investigating host-microbe interactions.

Phyllosphere microorganisms are crucial for plant and ecosystem health. They benefit host plants by improving nutrient uptake, increasing tolerance, protecting pathogens, regulating hormones, and degrading environmental toxins [12,13]. These plant–microbe interactions enhance ecosystem resilience and sustainable plant growth [14,15,16]. Certain phyllosphere bacteria, such as *Microbacterium*, *Thermus*, and *Methylobacterium*, produce growth regulators like IAA and fix nitrogen, improving plant growth and nutrition [17,18]. Atmospheric dinitrogen fixed by genera, like *Beijerinckia* and *Azotobacter*, can be absorbed by leaves or transported to roots, affecting plant growth [19]. In rice, interleaf microbes fix nitrogen and produce antimicrobial substances, indirectly promoting plant growth [20]. Phyllosphere microorganisms contribute to nutrient cycling and influence microbial communities in soil, water, and air, with implications for conservation and restoration efforts [21,22,23]. This emerging field has broad implications for plant and ecosystem health, sustainable agriculture and land use management.

Phyllosphere microorganism sources are complex, influenced by both biotic factors (host characteristics, insect activity, and microbial interactions) and abiotic factors (soil, climate, and temperature) [4,11,24,25,26]. Plant-emitted volatile organic compounds significantly shape phyllosphere microbial communities, serving as antimicrobial agents or carbon sources [27,28]. For instance, *Methylobacterium* and *Candida boidinii* can utilize methanol as a growth substrate [27,29]. Plant volatiles also contain fungicidal substances that help plants resist pest stress and impact microbial community structure. Understanding leaf VOCs can provide insights into phyllosphere community composition and plant–microbe interactions.

Global trade and cultural exchanges have led to the introduction of exotic species, some becoming invasive and damaging natural ecosystems. *S. trilobata* (L.) Pruski, a South American perennial herb introduced to China in the 1970s, is now one of the world’s 100 most noxious invasive species. In southern China, it has displaced the native *S. calendulacea*. Recent studies on *S. trilobata* have focused on its ecological impacts [30], phytochemical properties [31], and eco-physiological traits [32,33]. 

While many studies have investigated the invasion mechanism of *S. trilobata*, focusing on chemosensory effects and stress response [34,35], limited research exists on its phyllosphere microbial communities and growth-promoting VOCs. Endophytic bacteria and leaf-emitted VOCs have been shown to facilitate *S. trilobata’s* growth and competitive advantages [36,37,38], suggesting that both microbial community structure and secondary metabolites may contribute to its successful invasion. To investigate the invasion mechanism of *S. trilobata*, we studied the invasive plant, its native counterpart *S. calendulacea*, and their hybrids. We used Illumina NovaSeq high-throughput sequencing of the 16S rRNA V3-V4 fragment to analyze phyllosphere microbiome formation, succession, and development. Additionally, we used headspace solid-phase microextraction (HS-SPME) coupled with gas chromatography mass spectrometry (GC-MS) to identify the composition and proportion of volatile compounds in *Sphagneticola* species. Our research aims to explore the relationship between phyllosphere microbial composition and volatile compounds, providing insights into the invasion mechanisms of *S. trilobata*.

## 2. Materials and Methods

### 2.1. Sample Preparation

In March 2022, we collected *S. calendulacea*, *S. trilobata*, and their F1 hybrids from the field in Beidou Town, Taishan City, Guangdong Province (21.879192° N, 112.410689° E). For each species/taxon, we randomly selected 15 uniform growing samples. For each sample, we collected two intact, healthy leaves at the same height using sterilized scissors and stored them in sterile 50mL conical tubes. All leaf samples were stored at −80 °C until assay analysis. 

### 2.2. DNA Extraction

We extract phyllosphere microbiome DNA following methods from [39,40]. Briefly, we weighed 10 g of the leaf samples, cut them into pieces, and immersed them in sterile TE buffer (10 mmol/L Tris-HCl, 1 mmol/L EDTA, Ph 8.0) at a 1:20 ratio (leaf weight/buffer volume). The material was sealed with a sterilized membrane and shaken at 200 r/min for 30 min (HY-4, Ruihua Changzhou, China) at room temperature to separate microbial cells from the leaf surface. The mixture was then sonicated at 40 kHz for 15 min (JP-010T, Jiemeng, Shenzhen, China). Microorganisms were collected onto a 0.22 µm filter membrane using vacuum filtration in a sterile environment. Total DNA was extracted from filters using a FastDNA spin kit (Qbiogene, Irvine, CA, USA) per manufacturer’s instructions. Purified DNA was dissolved in 100 µL of ddH_2_O and stored at −20 °C.

### 2.3. PCR Amplification, Sequence Processing and Analysis

Sample DNA was diluted to 1 ng/µL with sterile water. The V3-V4 region of 16S rRNA was amplified using bacterial specific primers 515F (5′-GTGCCAGCMGCCGCGGTAA-3′) and 806R (5′-GGACTACHVGGGTWTCTAAT-3′). The PCR reaction mixture contained 15 µL 2× Phusion Master Mix, 3 µL each of forward and reverse primers (2 µmol/L), 7 µL genomic DNA (1 ng/µL), and 2 µL ddH_2_O. PCR reaction conditions were the following: 98 °C for 1 min; 30 cycles of 98 °C for 10 s, 50 °C for 30 s, and 72 °C for 30 s; and final extension at 72 °C for 5 min. Equal amounts of PCR productions were mixed, homogenized, and purified. Paired-end sequencing of 16S rRNA hypervariable regions was performed using the Illumina NovaSeq platform (Illumina, San Diego, CA, USA).

Raw sequencing data were analyzed using the QIIME2.0 DADA2 and USEARCH pipelines. After assembly, filtering, and quality control, amplicon sequence variants (ASVs) were defined at 100% similarity. Bacterial sequences were classified using the SILVA database (Release 138, http://www.arb-silva.de accessed on 12 May 2022). ASVs and basic analysis results were obtained for each sample, with composition structure statistics and diversity analysis conducted at various taxonomic levels.

### 2.4. Sample Prepration and Treatment for Volatile Compounds Detection

Harvested materials were immediately weighed, frozen in sterile 50 mL conical tubes, and stored at −80 °C. One gram of sample powder was transferred to a 20 mL headspace vial (Agilent, Palo Alto, CA, USA) containing NaCl-saturated solution to inhibit enzyme reactions. Vials were sealed with crimp-top caps using TFE-silicone headspace septa (Agilent Technologies, Santa Clara, CA, USA). For the solid-phase microextraction (SPME) assay [41], each vial was heated to 60 °C for 5 min; then, a 120 µm DVB/CWR/PDMS fiber (Agilent Technologies, Santa Clara, CA, USA) was exposed to the sample’s headspace for 15 min at 100 °C. 

### 2.5. GC-MS Conditions 

After sampling, we desorbed VOCs from the fiber coating in the GC injection port (Model 8890; Agilent, Santa Clara, CA, USA) at 250 °C for 5 min in splitless mode. VOCs were identified and quantified using an Agilent Model 8890 GC and a 7000D mass spectrometer (Agilent, Santa Clara, CA, USA), equipped with a 30 m × 0.25 mm × 0.25 μm DB-5 MS capillary column (5% phenyl-polymethylsiloxane). Helium served as the carrier gas at 1.2 mL/min. The injector and detector temperatures were 250 °C and 280 °C, respectively. Oven temperature was programmed from 40 °C (3.5 min), increasing to 100 °C at 10 °C/min, to 180 °C at 7 °C/min, to 280 °C at 25 °C/min, and held for 5 min. Mass spectra were recorded in electron impact (EI) ionization mode at 70 eV. Quadrupole mass detector, ion source, and transfer line temperatures were set at 150 °C, 230 °C, and 280 °C, respectively. We used selected ion monitoring (SIM) mode for analyte identification and quantification.

### 2.6. Bioinformatics and Statistical Analysis

Analyses were performed in R 3.4.3. We filtered out ASVs classified as chloroplasts and mitochondria. α-diversity was estimated using Chao1, Shannon’s and Simpson’s indices on absolute abundance matrices. ANOVA was used to analyze significant differences for those indices among three *Sphagneticola* species, with Tukey’s honest significant difference test for post-hoc comparisons. Relationships among bacterial community structures of the three *Sphagneticola* species were assessed using principal coordinate analysis (NMDS) based on Bray–Curtis distances and UPGMA clustering tree structures.

## 3. Results

### 3.1. Varied Species Richness and Diversity of Phyllosphere Microbes among Two Sphagneticola Species and Their F1 Hybrids

**Figure 1:**
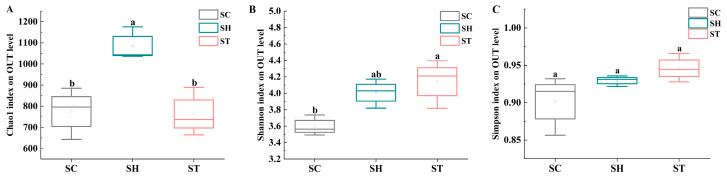
α-diversity indices for the 16S rRNA gene sequences. Box plots of the Chao1 (**A**), Shannon (**B**), and Simpson (**C**) indices in phyllosphere samples from both *S. calendulacea* (SC), *S. trilobata* (ST) and their F1 hybrids (SH). Whiskers represent the minimum and maximum values. The bar represents the median. Bonferroni correction-adjusted p-value was calculated from Dunn’s test of multiple comparisons using rank sums. The different letters represent significant differences between groups (*p* < 0.05).

High-throughput sequencing of *S. trilobata*, *S. calendulacea* and their F1 hybrid yielded 3,418,736 raw sequences, with 3,413,498 high-quality sequences retained after filtering. Rarefaction curves indicate sufficient sequencing depth (Figure S1). α-diversity analyses revealed the following: ➀ Chao1 index: F1 hybrids (1082.00 ± 78.89) showed significantly higher species richness than both parents. No significant difference was observed between *S. calendulacea* (774.76 ± 122.40) and *S. trilobata* (763.33 ± 114.79) (Figure 1A). ②. Shannon index: *S. trilobata* (4.14 ± 0.30) exhibited significantly higher diversity than *S. calendulacea* (3.60 ± 0.13) (Figure 1B). The F1 hybrid (4.01 ± 0.18) did not differ significantly from either parent (Figure 1B). ③. Simpson index: No significant differences were found among *S. calendulacea* (0.90 ± 0.04), *S. trilobata* (0.95 ± 0.02), and the F1 hybrid (0.93 ± 0.01) (Figure 1C).

### 3.2. Distinct Phyllosphere Microbial Community Structure at Family and Genus Levels among Three Sphagneticola Groups

**Figure 2:**
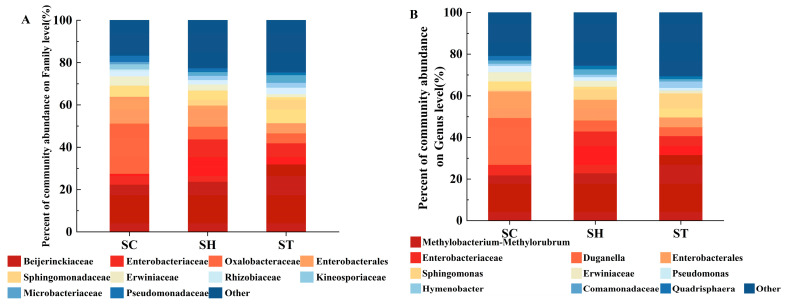
Average relative abundances of bacterial at the family level (**A**) and the genus level (**B**) in the different species. SC, *S. calendulacea*; SH, the hybrids; ST, *S. trilobata*.

Taxonomic annotation revealed significant differences in community structure between the host plant groups at both the family and genus levels. Taxa with < 0.01 relative abundance were grouped as “others”. At the family level, 10 bacterial families dominated the phyllosphere communities, comprising > 50% of relative abundance (Figure 2A). The family *Beijerinckiaceae* was most abundant on *S. trilobata* (32.0%) and the hybrid (23.7%), but lower on *S. calendulacea* (22.4%). In contrast, the family *Oxalobacteraceae* was most prevalent on *S. calendulacea* (23.8%) but rarer on *S. trilobata* (4.8%) and the hybrid (5.9%). At the genus level (Figure 2B), *Methylobacterium-Methylorubrum* dominated on *S. trilobata* (31.7%) and the hybrid (22.9%), but were less abundant on *S. calendulacea* (21.9%). *Duganella* was most prominent on *S. calendulacea* (22.5%) but rarer on *S. trilobata* (4.3%) and the hybrid (5.3%). While the hybrid’s microbial community was intermediate between its parents at the family level, it more closely resembled *S. trilobata* at the genus level.

### 3.3. Distinct Phyllosphere Microbial Community Assemblies among Three Sphagneticola Species Groups

**Figure 3:**
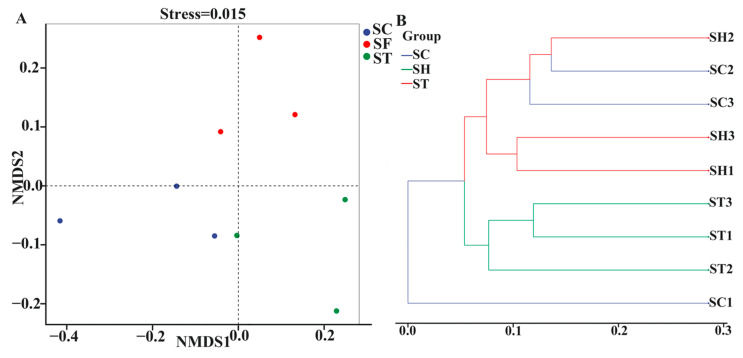
Principal coordinate analysis (NMDS) (**A**) and UPGMA clustering analysis (**B**) of the bacterial communities in different species based on Bray–Curtis distances. SC: *S. calendulacea*; SH: the hybrids; ST: *S. trilobata*.

β-diversity analyses compared overall bacterial community compositions between host plant groups. Non-metric multidimensional scaling (NMDS) and UPGMA clustering both revealed significantly distinct phyllosphere community structures among *S. trilobata*, *S. calendulacea* and their hybrid. NMDS analysis separated samples into three mutually exclusive groups (Figure 3A). *S. trilobata* clustered alone in Group 1, while *S. calendulacea* and the hybrid formed separate clusters in Groups 2 and 3, respectively. UPGMA clustering showed a slightly different pattern. *S. trilobata* remained in a distinct cluster, but *S. calendulacea* and hybrid communities intermingled, suggesting higher similarity and shared bacterial membership.

One *S. calendulacea* sample (SC1) formed an outgroup branch, indicating the most dissimilar community. This outlier may result from localized environmental variability or stochastic microbial association. The remaining *S. calendulacea* and hybrid communities grouped together with interspersed branches, reflecting an intermediate state between complete differentiation and homogeneity.

### 3.4. Distinct Profile of Volatile Organic Compounds among Three Sphagneticola Species

*S. calendulacea* contained the most diverse VOCs profile, with 72 total compounds detected, eighteen comprising > 1% relative abundance (Table 1). The hybrid and *S. trilobata* contained 60 and 73 VOCs, respectively, with 18 and 13 at > 1% relative abundance (Table 1). While all groups were dominated by terpenes and aromatics, *S. calendulacea* also had more esters and alcohols, suggesting niche differentiation in their VOC profiles.

The native *S. calendulacea* exhibited a distinct VOC profile, including styrene, p-xylene, cis-muurola-4(14),5-diene, and benzyl benzoate, with β-cubebene as the most abundant compound (14.39%; Table 1). The hybrid was characterized by copaene, β-cis-ocimene, 1,4-cadinadiene, α-muurolene, and α-cadinene, with (E)-germacrene D predominant (24.59%; Table 1). While sharing some VOCs with *S. calendulacea*, such as β-cubebene, the hybrid’s profile was intermediate yet distinct, with unique compounds and different relative quantities. *S. trilobata* displayed the greatest differentiation, containing several unique VOCs including p-cymene, (1S)-(—)-α-pinene, β-ocimene, cis-muurola-3,5-diene, and phenylethyl alcohol. Its major VOC was α-phellandrene (20.37%; Table 1). The presence of multiple unique VOCs and differences in the relative abundances of shared compounds demonstrates that *S. trilobata’s* volatile signature was the most divergent among the three groups.

**Figure 4:**
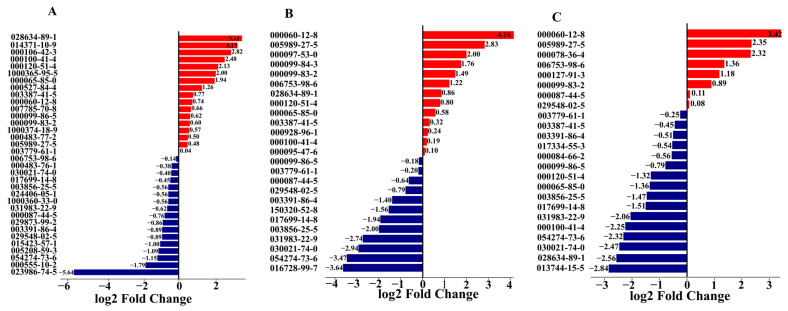
Comparison of differences between SC and SH (**A**), ST and SH (**B**), SC and ST (**C**) volatile compounds based on fold change. SC: *S. calendulacea*; SH: the hybrids; ST: *S. trilobata*. Red and blue bar indicates upregulation and downregulation respectively.

Fold change analysis quantified VOC differences among *S. calendulacea*, *S. trilobata* and their hybrid. *S. calendulacea* upregulated primarily hydrocarbons and aldehydes compared to the hybrid, with 2-thujene showing the greatest increase (3.41-fold; Figure 4A). Conversely, (E)-germacrene D was downregulated 5.64-fold in *S. calendulacea* relative to the hybrid. Comparing the hybrid and *S. trilobata* revealed distinct differences, with phenylethyl alcohol and D-limonene upregulated 4.16-fold in *S. trilobata*, while terpenes and aromatics were downregulated (Figure 4B). *S. trilobata* had fewer upregulated VOCs compared to *S. calendulacea* but showed an increase in phenylethyl alcohol (3.42-fold), D-limonene (2.35-fold) and humulene (1.36-fold), while β-cubebene decreased 2.82-fold (Figure 4C). β-cubebene’s abundance in *S. calendulacea* indicates its volatile signature was most differentiated from *S. trilobata*.

However, in the absence of data on the ecological roles and impacts of these specific VOCs, further work is required to determine whether the differentiation in volatile signatures observed here provides a fitness advantage. Analyzing VOC profiles from additional populations of native, introduced and hybrid *Sphagneticola*, especially in areas of introduction and hybridization, would provide a broader understanding of how volatile evolution shapes diverse ecological interactions within and outside a plant’s native range.

## 4. Discussion

### 4.1. Divergent Diversity and Assembly of Phyllosphere Microbial Communities among Sphagneticola Species and Their Hybrids

α-diversity indices (Chao1, Shannon, and Simpson) revealed higher species richness and diversity in *S. trilobata’s* phyllosphere microbial communities compared to *S. calendulacea*. The F1 hybrid exhibited comparable diversity and evenness to both parent groups, suggesting inheritance of a diverse, well-distributed assortment of microbial species, which may influence ecological interactions and adaptation [42].

β-diversity analyses (NMDS and UPGMA clustering) showed distinct bacterial community structures among *S. trilobata*, *S. calendulacea*, and the hybrid. While *S. trilobata* consistently formed a separate group, *S. calendulacea* and the hybrid displayed some shared membership, indicating heterogeneity or intermediate character in their assembly. This supports the influence of plant genotype, environmental heterogeneity, and microbial dispersal on plant–microbe interactions [2,4,11,24,26]. Further research is needed to understand the drivers of both differentiation and occasional convergence in these phyllosphere communities [43].

These findings have potential ecological and evolutionary implications. *S. trilobata’s* higher microbial diversity may provide a competitive advantage over native species, offering a broader range of functional traits and potential benefits [44]. The shared membership between *S. calendulacea* and the hybrid suggests certain microbial taxa are well suited to colonize both native and hybrid hosts, possibly due to adaptability to various environmental conditions or host genotypes [45].

Understanding the complex interactions between plants and their associated microbial communities is crucial for predicting the ecological and evolutionary consequences of these relationships. For example, the higher species richness and diversity in *S. trilobata* may contribute to its success as an invasive species. A diverse microbiome can enhance plant resilience to environmental stressors and promote nutrient acquisition [11,44]. Furthermore, the shared microbial membership between *S. calendulacea* and the hybrid may facilitate gene flow between native and introduced species, potentially leading to the emergence of novel genotypes and phenotypes [42].

### 4.2. Chemosensitive Compounds and Invasive Success: The Role of Volatile Composition in S. trilobata’s Dominance Over Native Plant Species

Exotic species generate chemosensitive compounds that hinder the growth of indigenous plants through the volatilization of stems and leaves and the secretion of roots [37,38]. Infusions from various parts of *S. trilobata* negatively affect common crops like rice and tomato [46,47]. The volatile composition of *Sphagneticola* species differs, and examining their phyllosphere VOCs can shed light on the potent chemosensory effects of *S. trilobata*. Terpenoids, prevalent in the phyllosphere volatiles of the three *Sphagneticola* species, possess excellent fungicidal properties and are commonly used in fungicides [48].

*S. trilobata* has fewer VOCs with a relative content above 1% compared to native species and hybrids, but it has a higher relative content of compounds with fungicidal effects, such as α-Phellandrene and Caryophyllene. The unique compound p-cymene, found in *S. trilobata*, has a relative content of 13.33%, second only to α-Phellandrene and D-Limonene among all volatiles. p-Cymene exhibits various pharmacological properties, including antibacterial [49], antiparasitic [50], and antiviral activities [51]. Fold-change analysis reveals that the most downregulated volatile in *S. trilobata* compared to *S. calendulacea* and their hybrids is phenylethyl alcohol. This compound, which has an aromatic odor, is known to inhibit a range of pathogenic bacteria [52].

This evidence suggests that *S. trilobata* contains more bacteriostatic volatile substances than *S. calendulacea* and the hybrids. Studies have shown that the growth performance of *S. trilobata* supplemented with its own extract is superior to that of *S. calendulacea* in any cultivation environment, indicating that the VOCs of invasive plants can inhibit other plants without causing autotoxicity [53,54]. These antibacterial and anti-pest VOCs can help protect *S. trilobata* from most pests and diseases during its invasion, providing it with a competitive advantage over native plants. The stronger chemosensitization of *S. trilobata* compared to *S. calendulacea* and their hybrids further contributes to its ability to outcompete and suppress the growth of surrounding native plants.

In conclusion, the unique volatile composition of *S. trilobata*, including compounds like p-cymene, phenylethyl alcohol, α-Phellandrene, and Caryophyllene, may play a critical role in its invasive success. These chemosensitive substances not only inhibit the growth of native plants but also provide protection against pests and diseases, allowing *S. trilobata* to thrive in various environments and outcompete other plant species.

### 4.3. Volatile-Mediated Plant–Microbe Interactions among the Sphagneticola Species

Plant leaves emit VOCs that can influence the composition of their phyllosphere microbial communities. Compounds like terpenoids, phenylpropanoids and phencylides have antimicrobial properties and can inhibit colonization [55,56]. *S. trilobata* produces high amounts of terpenoids with insecticidal and antimicrobial activity, which may contribute to its lower microbial diversity relative to the other *Sphagneticola* groups.

The VOCs from *S. trilobata* could interfere with native plants and microbes, enabling the formation of single-species communities. Antifungal VOCs may also allow *S. trilobata* to avoid native pathogens, facilitating invasion success. However, microbial communities can reciprocally shape the production of plant VOCs. For example, *Bacteroides*, *Bacillales* and *Methylobacterium*-*Methylorubrum*, found on raspberries, produce VOCs that increase pest/disease resistance [57,58].

In this study, *Methylobacterium*-*Methylorubrum* was most abundant across all *Sphagneticola* groups, especially the hybrid. This suggests the hybrid, not just *S. trilobata*, may possess strong chemical effects that reduce suitable habitat for native species. Plant–microbe interactions and their shared chemical mediators warrant further study as determinants of community structure and potential drivers of species invasion.

The reciprocal relationships between plants, microbes and their volatile signals are complex but ecologically significant. While terpenoid-rich *S. trilobata* may chemically inhibit competitors to dominate communities, associated microbes like *M. methylorubrum* could simultaneously enhance its defenses and stress tolerance in new ranges. However, whether the high abundance of *M. methylorubrum* provides benefits, and what microbial-derived VOCs are involved, requires experimental confirmation. The hybrid’s combined volatile chemistry and microbial profile may represent an intermediate or novel state that similarly influences community assembly and species distributions.

Overall, volatile-mediated plant–microbe interactions deserve greater consideration as mechanisms not only promoting but also potentially limiting or regulating the spread of invasive species outside their native ranges. *S. trilobata*’s chemical effects on and associations with other organisms likely depend on attributes of both its new and ancestral environments, as well as its resident microbiota. The hybrid’s unique characteristics may also derive from a combination of introduced and native influences that shape its biological and ecological relationships in a distinct manner. Further work could explore how terpenoid profiles, microbial communities and their interactive VOCs differ quantitatively and qualitatively between these groups across native and introduced populations. Manipulative experiments are also needed to determine the causal roles of plant and microbial volatiles in community dynamics, species coexistence and habitat generation or loss. By integrating chemical and biological approaches, deeper insights may be gained into the complex factors underlying plant invasion, competitive exclusion of natives, and conservation of diversity even between closely-related species.

## 5. Conclusions

In this study, we examined the phyllosphere microbial community structure and VOCs in three *Sphagneticola* species: the introduced *S. trilobata*, the native *S. calendulacea* and their hybrid. Despite their close relationships, these species exhibit distinct microbial communities, volatile chemistries and ecological characteristics that together may determine their spread and competitive ability.

Specifically, firstly, *S. trilobata* harbors higher microbial diversity and produces more potent antimicrobial volatiles, likely facilitating its invasion success and dominance in new environments. However, the presence of certain shared microbes among the three groups indicates a shared resilience in coping with similar environment stresses. Secondly, the hybrid’s intermediate traits warrant further study as potential drivers of habitat generation, genetic exchange, or modulation of chemical effects on community structure. Thirdly, volatile-mediated plant–microbe interactions influence invasion, competitive exclusion, and coexistence. These interactions depend on attributes of both ancestral and new ranges as well as resident organisms. Finally, an interdisciplinary approach combining chemical and microbial ecology is needed to understand plant–microbe dynamics at fine scales, which may have significant consequences for species distributions and ecosystem properties. Findings could reveal opportunities for novel control strategies or harnessing positive interactions for diversity conservation in a changing world.

This system provides a tractable model to advance understanding of mechanisms that generate and maintain biodiversity—or lead to its loss. By examining closely-related native, introduced, and hybrid groups across ranges, we can gain new insights into the complex relationships shaping community assembly at the invasion front. These findings could inform novel control strategies or methods to harness positive interactions for diversity conservation in changing environments.

## Figures and Tables

**Table 1:** Composition of volatile compounds in *S. calendulacea*, *S. trilobata* and their hybrids.

	*S. calendulacea*			F1			*S. trilobata*		
Volatile Component	CAS Number	Classification	Content (%)	CAS Number	Classification	Content (%)	CAS Number	Classification	Content (%)
β-cubebene	013744-15-5	Terpenoids	14.39	/	/	/	013744-15-5	Terpenoids	2.07
α-phellandrene	000099-83-2	Terpenoids	11.04	000099-83-2	Terpenoids	7.27	000099-83-2	Terpenoids	20.37
(1R)-(+)-α-pinene	007785-70-8	Terpenoids	10.05	007785-70-8	Terpenoids	6.38	/	/	/
2-thujene	028634-89-1	Hydrocarbons	7.55	/	/	/	028634-89-1	Hydrocarbons	1.29
β-caryophyllen	000087-44-5	Terpenoids	6.67	/	/	/	/	/	/
δ-cadinene	000483-76-1	Terpenoids	6.51	/	/	/	/	/	/
o-cymene	000527-84-4	Terpenoids	4.95	000527-84-4	Terpenoids	2.06	/	/	/
sabenene	003387-41-5	Terpenoids	3.97	003387-41-5	Terpenoids	2.32	003387-41-5	Terpenoids	2.91
γ-elemene	029873-99-2	Terpenoids	3.16	029873-99-2	Terpenoids	5.7	/	/	/
styrene	000100-42-5	Aromatics	3.11	/	/	/	/	/	/
D-limonene	005989-27-5	Terpenoids	2.89	005989-27-5	Terpenoids	2.08	005989-27-5	Terpenoids	14.74
γ-muurolene	030021-74-0	Terpenoids	2.57	030021-74-0	Terpenoids	3.36	/	/	/
humulene	006753-98-6	Terpenoids	1.74	006753-98-6	Terpenoids	1.91	006753-98-6	Terpenoids	4.45
p-xylene	000106-42-3	Aromatics	1.41	/	/	/	/	/	/
(+)-epi-bicyclosesquiphellandrene	054274-73-6	Aromatics	1.27	/	/	/	/	/	/
β-phellandrene	000555-10-2	Terpenoids	1.12	000555-10-2	Terpenoids	3.91	/	/	/
cis-muurola-4(14),5-diene	1000365-95-5	Hydrocarbons	1.04	/	/	/	/	/	/
benzyl benzoate	000120-51-4	Esters	1.01	/	/	/	/	/	/
(E)-germacrene D	/	/	/	023986-74-5	Terpenoids	24.59	/	/	/
caryophyllene	/	/	/	000087-44-5	Terpenoids	11.29	000087-44-5	Terpenoids	7.2
γ-cadinene	/	/	/	000483-76-1	Terpenoids	8.49	/	/	/
1,2,3,4,4a,5,6,7-octahydro-4-methyl-7-methylene-1-(1-methylethyl)-, (1S,4R,4aS)-Naphthalene	/	/	/	054274-73-6	Aromatics	2.81	/	/	/
copaene	/	/	/	003856-25-5	Terpenoids	1.18	/	/	/
β-cis-ocimene	/	/	/	003338-55-4	Terpenoids	1.16	/	/	/
1,4-cadinadiene	/	/	/	016728-99-7	Terpenoids	1.11	/	/	/
α-muurolene	/	/	/	031983-22-9	Terpenoids	1.1	/	/	/
α-cadinene	/	/	/	024406-05-1	Terpenoids	1.04	/	/	/
p-cymene	/	/	/	/	/	/	000099-87-6	Aromatics	13.33
(1S)-(-)-α-pinene	/	/	/	/	/	/	007785-26-4	Terpenoids	10.82
β-ocimene	/	/	/	/	/	/	013877-91-3	Terpenoids	3.74
3,7,11,11-tetramethylbicyclo[8.1.0]2,6-undecadiene	/	/	/	/	/	/	067650-90-2	Terpenoids	2.95
cis-muurola-3,5-diene	/	/	/	/	/	/	1000365-95-4	Aromatics	1.69
phenylethyl alcohol	/	/	/	/	/	/	000060-12-8	Alcohols	1.07

## Data Availability

The raw sequence data supporting these findings were submitted to the NCBI Sequence Read Archive (SRA) database (http://www.ncbi.nlm.nih.gov/ (accessed on 28 June 2023)), under BioProject accession number PRJNA988409.

## Supplementary Materials

The following supporting information can be downloaded at: https://www.mdpi.com/article/10.3390/genes15070955/s1, Figure S1: Sample rarefaction curves.
