# Supplementary figures and images for: Divergence of Phyllosphere Microbial Community Assemblies and Components of Volatile Organic Compounds between the Invasive Sphagneticola trilobata, the Native Sphagneticola calendulacea and Their Hybrids, and Its Implications for Invasiveness
Source: Genes (Basel). 2024 Jul 20;15(7):955. doi: 10.3390/genes15070955 (PMC11275861; doi:10.3390/genes15070955)

# Rarefaction Curves

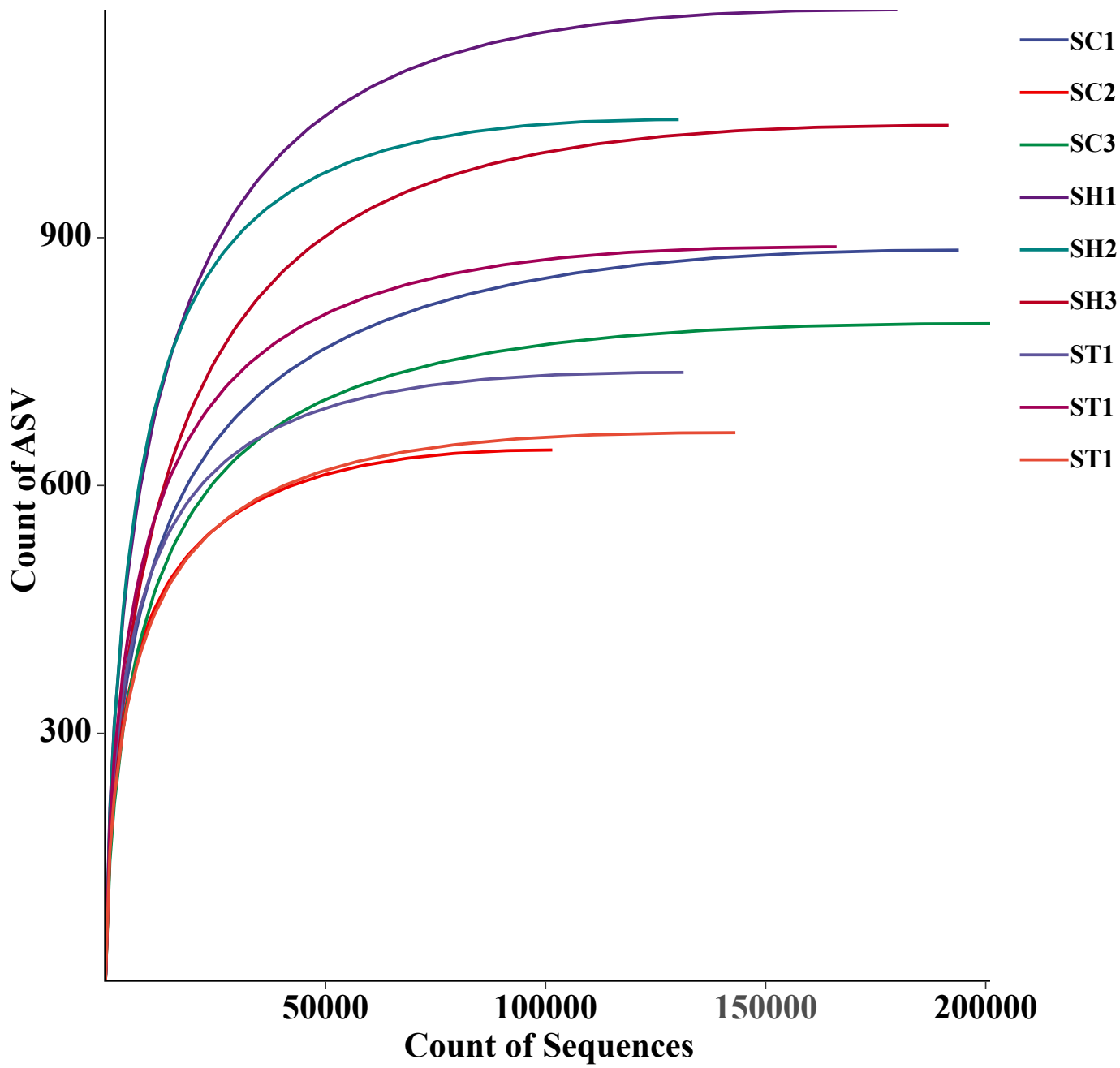

Supplement: Supplementary file 1 [file genes-15-00955-s001.zip › genes-3083683-supplementary.pdf]
